# From NAFLD to MAFLD: Definition, Pathophysiological Basis and Cardiovascular Implications

**DOI:** 10.3390/biomedicines11030883

**Published:** 2023-03-13

**Authors:** Andrea Boccatonda, Lorenzo Andreetto, Damiano D’Ardes, Giulio Cocco, Ilaria Rossi, Susanna Vicari, Cosima Schiavone, Francesco Cipollone, Maria Teresa Guagnano

**Affiliations:** 1 Internal Medicine, Bentivoglio Hospital, AUSL Bologna, 40010 Bentivoglio (BO), Italy; 2 Internal Medicine, IRCCS Azienda Ospedaliero-Universitaria Policlinico di Sant’Orsola, 40138 Bologna, Italy; 3 Department of Medicine and Aging Science, “Clinica Medica” Institute, ‘SS Annunziata’ Hospital, “G. d’Annunzio” University, 66100 Chieti, Italy; 4 Internistic Ultrasound Unit, SS Annunziata Hospital, “G. d’Annunzio” University, 66100 Chieti, Italy

**Keywords:** MAFLD, NAFLD, cardiovascular, diabetes, NASH

## Abstract

Non-alcoholic fatty liver disease (NAFLD) is defined as a chronic liver disease characterized by excessive fat accumulation in the liver without another obvious cause (no excessive alcohol consumption, hepatotoxic medications, toxins, viral infections, genetic hepatic diseases), therefore it is an exclusion diagnosis. The term NAFLD literally refers to non-alcohol related hepatopathy and does not adequately correlate with metabolic dysfunction and related cardiovascular risks. Therefore, researchers and scientific societies have moved towards changing the terminology. The novel nomenclature for a metabolic-associated fatty liver disease (MAFLD) has been proposed in 2020 by a group of experts to overcome the issues related to the old terminology. The diagnosis of MAFLD is based on the presence of hepatic steatosis and at least one between these three conditions: type 2 diabetes mellitus (T2DM), obesity or metabolic dysregulation. MAFLD has been shown to be an independent risk factor for cardiovascular diseases and atherosclerosis. It is better related to the main risk factors for atherosclerosis and cardiovascular diseases than NAFLD, such as dyslipidemia, T2DM and hypertension. The aim of this review is to highlight the reasons why the term NAFLD is moving to the term MAFLD, what are the conceptual basis of this choice and its clinical implications, particularly in the cardiovascular field.

## 1. Introduction: From NAFLD to MAFLD

Nowadays, the most common liver disorder is non-alcoholic fatty liver disease (NAFLD) [1,2] that affects 17–51% of adults worldwide [1,2,3]. The prevalence is steadily increasing and accompanies that of type 2 diabetes mellitus (T2DM) and obesity [4]. As the prevalence of NAFLD increases, it is assumed that the incidence of its complications (non-alcoholic steatohepatitis (NASH), decompensated cirrhosis, hepatocellular carcinoma (HCC), etc) will also increase progressively [5]. NAFLD is the second most common cause for liver transplantation, but it is quite probable that it will be soon the first one [6]. Despite awareness of the progressive increase in the incidence of that disease, there has been no significant progress in treatment and management of NAFLD in last years. 

NAFLD is defined as the present of fat in the liver on imaging and/or on liver biopsy, after exclusion of another obvious cause of liver damage (e.g., no excessive alcohol consumption, hepatotoxic medication, toxins, viral infections, genetic hepatic diseases) [1]. 

The inappropriate nomenclature of any disease exerts a relevant effect on both patients and physicians who must deal with it. It is difficult for patients to understand what disease they are suffering from, and the severity of it, when the terminology is based on a negative term. The term NAFLD literally refers to non-alcohol related hepatopathy and does not adequately correlate with metabolic dysfunction and related cardiovascular risks. Therefore, researchers and scientific societies have moved towards changing the terminology. In 2005, Loria et al. suggested to introduce positive criteria to define NAFLD [7]. In 2018, the European Liver Patient’s Association asked for a switch of terminology [8]. Eslam et al. in 2019 asked to find a more appropriate name to define this pathology [9].

The aim of this review is to highlight the reasons why the term NAFLD is moving to the term MAFLD, what are the conceptual basis of this choice and its clinical implications, particularly in the cardiovascular field.

## 2. Definition of MAFLD

**Figure 1:**
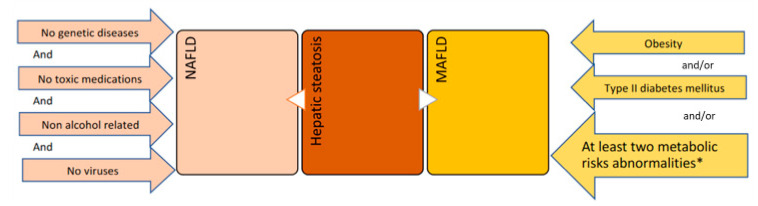
MAFLD vs. NAFLD diagnostic criteria. * Metabolic risk abnormalities: (1) Waist circumference > 102/88 cm in caucasian men/women or > 90/80 cm in Asian men/women; (2) Blood pressure ≥ 130/85 mmHg or antihypertensive medication; (3) Plasma Triglycerides > 150 mg/dl or triglycerides lowering drugs; (4) Plasma HDL-C < 40 mg/dl for men and < 50 mg/dl for women or specific lipid lowering drugs; (5) Prediabetes or HbA1c between 5.7–6.4%; (6) Plasma high-sensitivity C-reactive protein level > 2 mg/L.

The term MAFLD was proposed in 2020 by a group of researchers to overcome the issues related to the old terminology [9]. The diagnosis of MAFLD is based on the presence of hepatic steatosis (verified through imaging techniques and/or liver biopsy) and at least one between these three conditions: type 2 diabetes mellitus (T2DM), obesity and metabolic dysregulation (Figure 1). Metabolic dysregulation is defined as the presence of at least two metabolic risk abnormalities between [9]:

- Waist circumference ≥ 102/88 cm in Caucasian men/women or ≥ 90/80 cm in Asian men/women;
- Blood pressure ≥ 130/85 mmHg or antihypertensive medication;
- Plasma Triglycerides ≥ 150 mg/dl or triglycerides lowering medication;
- Plasma high-density lipoprotein cholesterol (HDL-C) < 40 mg/dl for men and < 50 mg/dl for women or lipid lowering medication;
- Prediabetes (fasting plasma glucose levels between 100–125 mg/dl or 2 h post load glucose levels between 140–199 mg/dl or glycosylated haemoglobin (HbA1c) between 5.7–6.4%;
- Homeostasis model assessment (HOMA) with insulin resistance score ≥ 2.5;
- High-sensitivity C-reactive protein levels > 2 mg/L.

## 3. MAFLD: Molecular and Pathophysiological Considerations

The prevalence of MAFLD is steadily increasing and may become the leading cause of chronic liver disease in the coming decades [10]. However, it is important to emphasize that this disease is a systemic disease. One of the complications is obesity, with a high prevalence in the developed countries. The prevalence of obesity has increased significantly over the past 40 years, from less than 3% in men and 6% in women in 1975 to 11% and 15% in 2016, respectively [10], making it a rapidly expanding public health concern.

There are multiple pathophysiological mechanisms associated with the development of obesity, ranging from neuroendocrine changes to lifestyle, food intake, reduced physical activity, genetics, and socio-economic factors. Changes over time in the balance between energy intake and energy expenditure can result in metabolic changes and dysfunction of white adipose tissue, leading to adipokine secretion, inflammation, and the development of chronic metabolic abnormalities. The liver is often implicated in obesity because it is the primary organ responsible for glucose and lipid metabolism. Bariatric surgery studies have demonstrated that MAFLD has been detected in 85–95% of severely obese individuals. Furthermore, meta-analysis have found that half of MAFLD patients and 82% of NASH patients are obese [11]. However, the liver is not the only organ in which metabolic abnormalities occur. These patients are at increased risk for extra- hepatic complications such as cardiovascular disease, chronic kidney disease (CKD), osteoporosis, obstructive sleep apnoea syndrome (OSAS), endocrine disorders, depression, and cognitive impairment [12,13,14].

The pathophysiology of MAFLD is complex and multifactorial. Some authors have proposed a pathophysiological model based on the "two-hit hypothesis" [15]. The first hit causes fat accumulation in hepatocytes, and the second hit causes oxidative stress, which increases inflammation and may lead to fibrosis in the long term [15]. The mechanism now appears to be more complex, and the "multiple hit" hypothesis is increasingly supported; MAFLD develops when triglyceride synthesis in the liver outweighs catabolism of non-esterified fats, and is dependent on oxidation in mitochondria and export of the same triglycerides to very low density lipoproteins (VLDL) [16]. Excess accumulation of fatty acids in the liver occurs through three pathways: lipolysis from adipose tissue, de novo lipogenesis and excessive intake of fat and sugar from the diet. In MAFLD patients, the expression of acetyl-coenzyme-A carboxylase 1 (ACC1), the first enzyme in de novo lipogenesis, is decreased and Acetyl-coenzyme-A has been shown to be converted to malonyl-CoA. Furthermore, accumulation of malonyl-CoA inhibits carnitine palmitoyl transferase (CPT)-1, which transports fatty acids within mitochondria and decreases β-oxidation. Fatty acid synthase (FAS) induces the conversion of malonyl-CoA to palmitic acid, and its expression is increased in patients with simple steatosis, while its function is reduced inpatients with NASH [17]. Thus, the mechanism leading to decreased triglyceride synthesis is found to be the accumulation of harmful free fatty acids.

NASH also shows an increase in free cholesterol in hepatocytes, stimulating inflammation and fibrosis in experimental models [17,18]. Both sterol SREBP-2 and HMG-CoA are increased in NASH patients. SREBP-2 is regulates HMG-CoA, an enzyme that limits cholesterol synthesis [19,20]. Thus, NASH is characterized by low HDL-cholesterol, high triglycerides and an increase of low-density lipoprotein (LDL)-C [21].

Recent studies have shown that non-parenchymatous liver cells, mainly liver sinusoidal endothelial cells (LSECs), play a main role in the development of chronic hepatopathy [22,23]. In the context of adipose infiltration into liver parenchyma, these cells become dysfunctional [22,23]. This endothelial dysfunction leads to increased recruitment of systemic myeloid lineage cells that induce and amplify inflammation and cell damage [22]. As MAFLD progresses, the damage related to endothelial dysfunction also seems to worsen, leading to the development of complications related to hepatic inflammation and fibrosis, from hepatic microcirculation dysfunction to portal hypertension [22,23].

In the pathophysiological mechanisms involved in MAFLD and NASH should be considered the elevation of oxidant molecules, fibrogenic mediators as transforming growth factor beta-1 (TGF-β1), insuline-like growth factor (IGF-1) and endothelin-1, inflammatory mediators as C-reactive protein (CRP), interleukin (IL)-6 and tumor necrosis factor (TNF)-α and pro-coagulant factors as fibrinogen, factor VIII, and plasminogen activator inhibitor-1 [24]. High values of those markers are found in the pathophysiology of obesity. They may create dysregulation of adipose tissue function contributing to insulin resistance mechanisms [21].

Moreover MAFLD could constitute an important cause of HCC. In fact, NAFLD is already the most important emerging cause of hepatocellular carcinoma (HCC) in Western countries, such as US, France and UK. The global prevalence of NAFLD-related HCC is likely to further increase in parallel with increase of obesity prevalence. The incidence of HCC among patients with non-cirrhotic NAFLD is approximately 0.1 to 1.3 per 1000 patient-years and it is lower than the annual incidence of HCC in NASH cirrhosis patients that ranges from 0.5% to 2.6%, but the NAFLD is spreading in general population and so this could further raise HCC incidence in next future [25]. Some authors noted a 9% yearly increase in NAFLD-related HCC prevalence from 2004 to 2009 in US [26].

Furthermore, a meta-analysis of 19 studies and almost 170,000 individuals with NASH reported that the prevalence of NAFLD-related HCC in patients with NASH but without cirrhosis is approximately 38% compared with 14% for other liver diseases [27]. Another important point is that in western countries as Italy a changing scenario in HCC epidemiology has been shown: in particular there is a progressive increase of non-viral cases and, particularly, of “metabolic” and “metabolic + alcohol” HCCs [28]. Moreover, the prevalence of MAFLD-related HCC in Italy is rapidly increasing and it will soon represent the most part of patients with HCC. Nevertheless, despite a less favourable cancer stage at diagnosis, patients with MAFLD related-HCC seem to have a reduced cancer aggressiveness with a lower risk of HCC-related death [29].

## 4. What Link between MAFLD and Cardiovascular Events?

MAFLD is an independent risk factor for cardiovascular disease and atherosclerosis. MAFLD is associated with major risk factors for atherosclerosis and cardiovascular disease (CVD), including dyslipidaemia, T2DM and hypertension [16]. Several studies have been conducted to determine what associations exist. A large meta-analysis several studies involving 85,000 patients has shown a strong association between MAFLD and atherosclerotic plaque at the level of the carotid artery, arterial stiffness, coronary artery calcification and endothelial dysfunction [30]. Furthermore, an associated correlation between improvement in MAFLD and reduction in carotid artery atherosclerosis has been demonstrated [30]. Otherwise, worsening liver disease was associated with an increased risk of coronary and carotid artery disease [30]. In another cohort study in the United States, 3756 individuals were evaluated with coronary-enhanced computer tomography angiography, which showed a higher incidence of cardiovascular events in patients with MAFLD compared to controls without MAFLD [31]. 

Several pathophysiological mechanisms have been proposed to explain the increased cardiovascular risk in patients with MAFLD; they include changed lipid profile (atherogenic dyslipidaemia), insulin resistance, endothelial dysfunction, low-grade inflammation and many others [32]. 

Atherogenic dyslipidaemia is a typical alteration of the lipid profile related to an increase of cardiovascular diseases [33,34,35]. 

The increase in circulating TRL seems to depend on a reduction in lipoprotein lipase (LPL), which in turn is due to an increase in angiopoietin like protein (ANGPTL) 3 and ANGPTL4 activities [36,37]. Plasma levels of those two proteins are typically increased in obesity, T2DM and NAFLD [36,37]. The above mechanism and the alteration of hepatic TLR clearance (linked to apolipoprotein (apo)B) are two fundamental mechanisms underlying lipid accumulation within the hepatic parenchyma and are genetically associated with an increased risk of developing atherosclerosis [38]. In addition, HDL protein dysfunction has been documented in patients with NAFLD, with smaller dimensions and faster clearance in the circulating stream compared to healthy controls. As a result, the antioxidant and anti-atherosclerotic effects of HDL are reduced [39]. Furthermore, NAFLD is also associated with increased oxidized LDL levels after oral fat loading compared to healthy controls [40]. Postprandial lipid levels in NAFLD have been reported to affect hepatic TG levels and are an independent cardiometabolic risk factor [41,42].

Insulin resistance, associated with visceral obesity and increased body weight, and impaired glucose metabolism underlie MAFLD and the pathogenesis of CVD [43]. The consequence of insulin resistance is the persistence of high levels of insulin in the bloodstream, which leads to the maintenance of an unfavourable metabolism. This mechanism is characterised by an increase in circulating glucose and free fatty acids, stimulating the release of triacylglycerol from hepatocytes, which are inevitably associated with an increased risk of developing atherosclerotic disease [44].

Furthermore, insulin resistance increases de novo lipogenesis (DNL) in NAFLD [45]. One of the underlying mechanisms is the transcription of the SREBP-1c factor, which activates a series of enzymes implicated in lipogenesis [46]. Indeed, in NAFLD patients, DNL contributes to the synthesis of 26% of intrahepatic triglycerides, compared to 5% in healthy patients [47]. 

## 5. MAFLD and Complication of CVD

In patients with MAFLD, several studies have been performed regarding cardiovascular risk in specific districts, thus assessing what are the complications of CVD. A recent study evaluated 3,087,640 MAFLD patients and showed that 169,433 cardiovascular events over a follow-up period of 10.0 years [48]. On multivariable analysis, HR for CV events was 1.16 (1.15–1.18) in the overweight (OW)-MAFLD patients, 1.23 (1.20–1.27) for lean-MAFLD and 1.82 (1.80–1.85) for DMT2-MAFLD subjects [48]. Therefore, patients with lean-MAFLD or DMT2-MAFLD were characterized by a higher CVD risk than those with OW-MAFLD [48]. In the following paragraphs we will describe the association between MAFLD and ischaemic stroke, structural cardiac abnormalities, cardiac arrhythmias and coronary artery disease.

### 5.1. Ischemic Stroke

Several studies have shown that the metabolic changes seen in MAFLD lead to an increased risk of ischemic stroke [49]. Data from a Korean cohort study showed that the risk of stroke increases with increasing fatty liver index [50]. However, alanine transaminase, aspartate transaminase and γ-glutamyl transferase levels did not independently predict stroke-related risk [50]. A meta-analysis of nine case-control and cohort studies also reported that MAFLD was associated with a 2.3-fold increased risk of ischemic stroke, independent of conventional CV risk, obesity, and patients with type II diabetes [49]. In addition, a case-control study of 295 ischemic stroke patients and 1942 healthy subjects showed that the degree of fibrosis detected by elastography was independently associated with an increased risk of stroke [49]. More recently, in a cohort study of 80,000 Chinese subjects followed for an average of 10.3 years, it was observed that MAFLD severity assessed by ultrasonography was associated with a higher risk of future ischemic stroke, independent of other CV risks [51].

### 5.2. Structural Cardiac Abnormalities

The prevalence of wall thickening of left ventricle and myocardial mass is higher in patients with MAFLD [52]. The Framingham heart study enrolled 2356 patients and demonstrated that the presence of fat within the liver is associated with left ventricular mass, wall thickness, mitral peak velocity, and left ventricular filling pressures [53]. Another trial revealed that liver fibrosis in patients with MAFLD correlated with a worse outcome in patients with preserved ejection fraction heart failure (HFpEF) [54].

A recent trial showed that MAFLD patients display a higher risk for developing cardiac systolic and subclinical systolic dysfunctions, as well as diastolic dysfunction [55]. In that study, MAFLD subjects were characterized by lower levels of glycyl tyrosine, lysophosphatidylcholine (LPC) (18:2/0:0) and ceramide (Cer) (d18:0/23:0) in comparison with controls [55]. MAFLD patients presented a lower ventricular ejection fraction (LVEF), average global longitudinal strain (GLS) values and higher E/e’ ratio [55]. Eventually, decreased glycyl tyrosine levels have been significantly related to reduced LVEF, thus suggesting its evaluation as a biomarker for heart failure [55]. 

### 5.3. Cardiac Arrhythmias

Many studies have established an association between MAFLD and cardiac arrhythmias as atrial fibrillation (AF), ventricular arrhythmias and QTc prolongation. MAFLD is associated with structural cardiac remodelling that inevitably induces an increased risk of alterations in the conduction tissue of the heart. QTc prolongation in individuals with MAFLD and T2DM predisposes to an increased risk of sudden cardiac death [30]. The presence of MAFLD is also associated with the presence of pericardial fat, which is associated with a high prevalence of atrial fibrillation. A study of 260 patients found that increased thickness of the interventricular septum and left atrial stiffness index were associated with an increased incidence of atrial fibrillation in patients with MAFLD [56].

In the Framingham Heart Study in 3744 patients, 267 patients with elevated ALT and AST showed an increased risk of AF independently 10 years after diagnosis [52].

In addition, patients with MAFLD had an increased incidence of premature ventricular beats and non-sustained ventricular tachycardia [52]. Another retrospective study including T2DM and MAFLD showed an increased risk of heart block in patients with MAFLD [54].

### 5.4. Coronary Artery Disease (CAD)

Regarding the risk of developing CAD, studies have moved towards a comparison between patients with MAFLD and patients with metabolic syndrome [57]. In general, the presence of metabolic syndrome correlates slightly more with the risk of developing CAD than the presence of MAFLD [57]. However, when considering patients with type II diabetes, the risk of developing CAD is higher in patients with MAFLD [58,59]. In contrast, the presence of metabolic syndrome in patients with type II diabetes mellitus does not predict a higher incidence of CAD [59]. It has been shown that the association of MAFLD with T2DM is correlate with more severe insulin resistance, higher BMI and higher visceral adiposity index (VAI), compared to the association of T2DM and metabolic syndrome [57,58]. This research implies that physicians must pay more attention in patients with T2DM and concomitant MAFLD, as they are at higher CAD and cardiovascular risk. A recent study by Bessho et al. showed that patients with MAFLD are at higher risk of coronary artery calcification (CAC) (with a CAC score >100) than patients with NAFLD [60]. In particular, MAFLD patients with diabetes mellitus have a higher risk of CAC than other MAFLD groups [60].

Particularly, MAFLD subjects with diabetes displayed higher risk for CAC than the other groups of MAFLD [60]. Therefore, MAFLD was significantly correlated with subclinical atherosclerosis in the general population [60].

In another study performed on asymptomatic subjects who underwent health check-ups, patients with MAFLD or NAFLD were characterized by CAC score > 100, coronary artery disease and by higher 10-year cardiovascular risk than healthy subjects [61]. Otherwise, NAFLD alone (not MAFLD) was not related an increased coronary risk, compared to MAFLD [61]. Therefore, in asymptomatic individuals, the definition of MAFLD appears to be a better predictor of ASCVD risk compared with NAFLD [61].

Moreover, MAFLD was found to be a common clinician condition in hospitalized patients with acute coronary syndrome (ACS) and was related to impaired physical function [62]. The incidence of new clinical events in patients with ACS was higher in those displaying MAFLD and lower physical function [62].

Another recent study was conducted on 3306 patients with chronic coronary syndrome (CCS) with MAFLD [63]. Authors demonstrated that CCS patients with MAFLD had significantly lower event-free survival rate and increased major adverse CV events (MACE) risk (both *p* < 0.05) [63]. 

Another intriguing aspect concerns the gender difference. Pre-menopausal women have a lower incidence of MAFLD than men at the same age and considering equal cardiovascular risks, assuming a protective role of oestrogen towards fatty liver disease [64]. The presence of MAFLD correlates better in men with the risk of developing CAD [64].

### 5.5. High Blood Pressure

MAFLD is significantly related to high blood pressure (BP), especially with increase in systolic BP [65]. Moreover, MAFLD is a common comorbidity in hypertensive patients and is related to markers of cardiorenal risk, especially with uric acid [66]. In a recent study performed by Liu et al, individuals with liver fibrosis had significantly higher BP levels and hypertension prevalence than those without [67]. Moreover, liver fibrosis score was significantly related to BP levels [67]. In the 6-year longitudinal cohort of 3661 individuals with MAFLD without liver fibrosis, the incidence rates of liver fibrosis increased with increasing BP levels [67]. Despite these findings, there are currently no specific published studies on the role of antihypertensive drugs in patients with MAFLD.

## 6. Therapeutic Strategies

**Figure 2:**
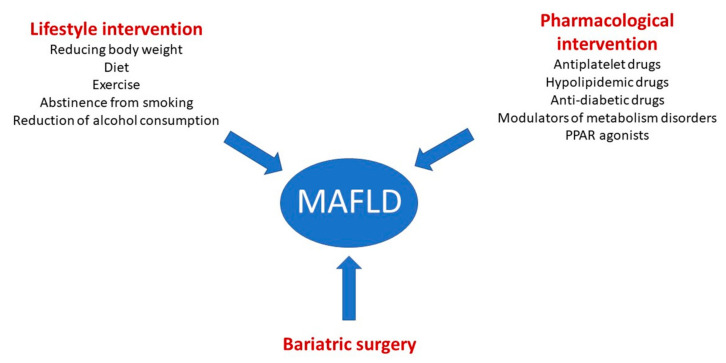
Graphic representation of principal therapeutic strategies in MAFLD.

The following therapeutic strategies on MAFLD are summarized in Figure 2 and principal ongoing trials are reported in Table 1.

### 6.1. Lifestyle Intervention

A key role in the treatment of MAFLD is lifestyle modification and the introduction of measures aimed at reducing body weight. Several studies have shown that a low-calorie diet has an impact on intrahepatic fat content [68,69]. Over the number of calories intake, the quality of food intake is also important. A low-glycaemic diet, rich in fruit and vegetables, and low in fat (especially saturated fat) is recommended. The prototype of this diet is represented by the Mediterranean diet, which is recommended for MAFLD patients [68,69]. Moreover, ketogenic diet seems also to exert a positive effect in reducing insulin resistance in MAFLD patients. However, further studies need to be performed regarding long-term effects and safety [70]. 

Another cornerstone in the treatment of MAFLD is exercise, both aerobic and resistance training [71,72]. One of the concerns encountered in overweight/obese patients with MAFLD, who undergo strict diets to decrease weight, is the loss of lean body mass with progressive development of sarcopenia. In that condition, resistance exercise aims to balance the reduction of calories intake, favouring a greater loss of fat mass and preserving lean body mass. Aerobic exercise has been shown to be poorly tolerated by overweight patients, resulting in poor compliance and effectiveness [73,74,75].

Diet in combination with exercise is intended to reduce body weight. A direct correlation between body weight reduction and histological improvement of fibrosis/steatohepatitis has been demonstrated [76].

Finally, abstinence from smoking and reduction of alcohol consumption simultaneously reduce the risk of MAFLD progression and the risk of developing cardiovascular disease [77].

### 6.2. Pharmacological Intervention

In patients with MAFLD, pharmacological/medical treatments aimed at reducing the amount of intrahepatic fat, stimulating metabolic processes, improving liver damage, maintaining low circulating lipid and glucose levels, and preventing cardiovascular events.

#### 6.2.1. Aspirin and Hypolipidemic Drugs

The use of acetylsalicylic acid is recommended in patients with established atherosclerotic disease [77]. Its use has been associated with the prevention of cardiovascular events and has been shown to reduce the risk of hepatic fibrosis [78]. Drugs aimed at reducing circulating and tissue-accumulated cholesterol and improving the lipid profile play an important role [79]. Treatment with statins and ezetimibe is recommended in patients with MAFLD because of their cardiological and hepatological advantages [77]. Proprotein convertase subtilisin/kexin type 9 (PCSK9) inhibitors are a new therapeutic option in patients with an unfavorable lipid profile despite maximal oral lipid-lowering therapy [77]. Studies have shown that this therapy is also safe in patients with liver disease [80]. 

#### 6.2.2. Anti-Diabetic Drugs

T2DM is often associated with MAFLD and is one of the most important risk factors for the development of this disease. Therefore, several studies are underway to demonstrate the efficacy of anti-diabetic drugs on fatty liver disease. 

A particularly well-studied class of drugs in this field are the glucagon-like peptide-1 (GLP-1) receptor agonists [81]. GLP-1 receptors are also present in human hepatocytes and are less represented in the hepatocytes of patients with MAFLD [82]. Studies initiated with exenatide, followed by liraglutide and semaglutide, have shown positive effects onweight loss, hepatic fibrosis and cardiovascular prognosis [81,83,84,85]. Semaglutide 2.4 mg has been approved by the Food and Drug Administration (FDA) of the US for the treatment of obesity [86].

New studies have been performed on sodium-glucose cotransporter 2 (SGLT2) inhibitors, now widely used for patients with high cardiovascular risk and T2DM. In a recent trial, empagliflozin demonstrated a histological improvement of hepatic steatosis, hepatocytes ballooning and fibrosis in patients with NASH and T2DM [87].

Thiazolidinediones (peroxisome proliferator-activated receptor (PPAR) agonists) were approved for patients with NASH demonstrated at liver biopsy and affected by T2DM [88]. There is still no strong evidence to recommend these drugs in patients with fatty liver at US or in patients without concomitant T2DM.

#### 6.2.3. Modulators of Metabolism Disorders

One of the main targets in the treatment of MAFLD is the reduction of lipid accumulation within hepatocytes. New studies have been performed on drugs activating the farnesoid X receptor [89,90,91]. This receptor is expressed in liver and intestinal cells and is involved in the synthesis and in the enteropathic circulation of bile salts [89]. Obeticholic acid (OCA) is the first of these drugs to achieve the primary endpoint of improving fibrosis in NASH patients. However, the drug’s approval has been delayed due to problems with side effects such as increased LDL, itching and reduced serum HDL [90]. Two other drugs are under investigation, EDP-305 and cilofexor [91]. In particular, the latter is not a bile acid and it seems to be associated with less side effects [91].

Another class of drugs under investigation are fibroblast growth factors (FGFs) 19 and 21 agonists that appear to improve the metabolic endocrine axis involving lipids, glucose and bile acids [92]. Promising studies have been performed on efruxifermin and pegbelferim, analogues of FGF 21. Both have been shown to be effective in reducing the amount of fat within the liver, monitored by magnetic resonance imaging proton density-fat fraction (MRI-PDFF). In addition, pegbelfermin showed an improvement in liver cytolysis indices and improved cardiometabolic parameters, while efruxifermin showed a benefit on lipid profile, glucose profile and weight loss [19,20].

#### 6.2.4. PPAR Agonists 

PPARs are ligand-activated transcription factors that modify lipid and glucose metabolism, regulating the body’s energy homeostasis. A distinction is made between PPARs alpha, which reduce plasma triglyceride levels, PPARs gamma, which regulate insulin secretion and lipid storage, and PPARs beta/delta, which increase fatty acid catabolism and reduce inflammation [93]. 

There are three groups of PPAR agonists: (1) PPAR α/δ is elafibranor (GFT505), (2) Pan-PPAR agonist (PPAR-α, PPAR- β/δ, and PPAR-γ) is lanifibranor (IVA337), and (3) Dual agonist of PPAR-α/γ is saroglitazar.

Lanifibranor is the first drug to meet the US FDA and European Medicines Agency (EMA) primary endpoints with statistically significant results; patients with NASH remission and one or more stages of fibrosis improvement will be used in histological analysis at week 72 as the primary composite endpoint [94]. In a phase IIB study, lanifibror was shown to be superior to placebo in terms of reducing fatty hepatitis (biopsy-proven) inpatients with NASH after 24 weeks of treatment [95].

Regarding saroglitazor, a multicentre phase II study was recently conducted that demonstrated an improvement in liver fat content measured by MRI after 16 weeks of treatment in patients with NASH [96]. 

Elafibranor reached phase III studies, discontinued early due to unproven efficacy of the drug after 72 weeks of treatment [97].

### 6.3. Bariatric Surgery

In obese or severely overweight patients, weight loss is not an easy goal to achieve. It has been documented that only 50% of obese patients succeed in decreasing their body weight by at least 7% in one year with lifestyle changes [76]. Bariatric surgery may be a possible therapeutic strategy, which has been shown to be effective in reducing drastically the body weight and improving steatohepatitis histologically. Given, however, the high pre- and post-operative risks in the short and long term, bariatric surgery is currently applied to those patients with BMI > 40 kg/m^2^ or BMI 35 to 40 kg/m^2^ with associated comorbid conditions [98]. A question on which there is still a lack of clarity is the safety of bariatric surgery in cirrhotic patients. Some meta-analyses demonstrate a low rate of post-operative complications in patients with cirrhosis evolved from MAFLD, however, adequate patient information and a meaningful evaluation of the risks and benefits of such procedures is always necessary [99,100].

Moreover, bariatric surgery can not only provide durable weight loss, but also effect on later development of HCC. In fact, at this regard, data of a recent meta-analysis have shown that bariatric surgery may be associated with a decreased risk of HCC [101].

## 7. Conclusions

Developed countries are witnessing an exponential increase in the prevalence of obesity and metabolic diseases, primarily diabetes. Screening for MAFLD is crucial in this context given the strong correlation and common aetiopathogenesis of these diseases. Notably, patients with MAFLD have a higher cardiovascular risk than patients without liver disease [102]. This awareness should increase the attention of clinicians towards patients with MALFD to try to prevent acute cardiovascular events that may lead to severe disability. The term MAFLD helps to provide more clarity regarding the etiopathogenesis and extrahepatic involvement of the disease itself. MAFLD is a disease that not only involves hepatologists but also brings other specialists into play. The term MAFLD will benefit the development of new endpoints for future drug trials. Furthermore, the detailed study of the etiopathogenesis of the disease, in particular regarding inflammation of the adipose tissue and lipid dysmetabolism may play an important role in unlocking the door to new treatment strategies in the future. 

Based on the definition and its pathophysiology, the monitoring of the MAFLD patient must be multidisciplinary. The evaluation of hepatic steatosis is based on ultrasound examination, along with the estimation of hepatic stiffness by using the shear wave methods. Furthermore, the contribution of the individual diabetes, nutritionist, cardiologist and vascular specialist must be managed within an integrated evaluation, ideally a multidisciplinary team.

## Figures and Tables

**Table 1:** Summary of ongoing clinical trials on new drug therapies for NASH resolution. FXR: Farnesoid X receptor; PPAR: Peroxisome proliferator-activated receptor; GLP-1: Glucagon-like peptide-1.

Drugs	Class	Trial	Phase	Trial ID
Obeticholic acid	FXR agonist 1st gen.	FLINT study	IIb	NCT01265498
		REGENERATE study (NASH with significant fibrosis)	III	NCT02548351
		REVERSE study (NASH with significant cirrhosis)	III	NCT03439254
Cilofexor	FXR agonist 2nd gen.	Cilofexor in patients with noncirrhotic NASH	II	NCT02854605
Elafibranor	PPAR α/δ agonist	GOLDEN- 505 study	IIb	NCT01694849
		RESOLVE- IT study	III	NCT02704403
Lanifibranor	Pan- PPAR agonist	NATIVE study	IIb	NCT03008070
Saroglitazar	Dual PPAR- α/γ agonist	EVIDENCE IV study	II	NCT03061721
Liraglutide	GLP-1 receptor agonist	LEAN study	II	NCT01237119
Semaglutide	GLP-1 receptor agonist	Subcutaneous semaglutide in NASH	II	NCT02970942

## Data Availability

Not applicable.
